# The Destructive Citrus Pathogen, ‘*Candidatus* Liberibacter asiaticus’ Encodes a Functional Flagellin Characteristic of a Pathogen-Associated Molecular Pattern

**DOI:** 10.1371/journal.pone.0046447

**Published:** 2012-09-28

**Authors:** Huasong Zou, Siddarame Gowda, Lijuan Zhou, Subhas Hajeri, Gongyou Chen, Yongping Duan

**Affiliations:** 1 United States Horticultural Research Laboratory, United States Department of Agriculture-Agricultural Research Service, Fort Pierce, Florida, United States of America; 2 Citrus Research and Education Center, University of Florida-Institute of Food and Agricultural Sciences, Lake Alfred, Florida, United States of America; 3 Indian River Research and Education Center, University of Florida, Fort Pierce, Florida, United States of America; 4 School of Agriculture and Biology, Shanghai Jiao Tong University, Shanghai, China; University of Wisconsin-Milwaukee, United States of America

## Abstract

Huanglongbing (HLB) is presently the most devastating citrus disease worldwide. As an intracellular plant pathogen and insect symbiont, the HLB bacterium, ‘*Candidatus* Liberibacter asiaticus’ (Las), retains the entire flagellum-encoding gene cluster in its significantly reduced genome. Las encodes a flagellin and hook-associated protein (Fla) of 452 amino acids that contains a conserved 22 amino acid domain (flg22) at positions 29 to 50 in the N-terminus. The phenotypic alteration in motility of a *Sinorhizobium meliloti* mutant lacking the *fla* genes was partially restored by constitutive expression of Fla*_Las_*. *Agrobacterium*-mediated transient expression *in planta* revealed that Fla*_Las_* induced cell death and callose deposition in *Nicotiana benthamiana*, and that the transcription of *BAK1* and *SGT1*, which are associated with plant innate immunity, was upregulated. Amino acid substitution experiments revealed that residues 38 (serine) and 39 (aspartate) of Fla*_Las_* were essential for callose induction. The synthetic flg22*_Las_* peptide could not induce plant cell death but retained the ability to induce callose deposition at a concentration of 20 µM or above. This demonstrated that the pathogen-associated molecular pattern (PAMP) activity of flg22 in Las was weaker than those in other well-studied plant pathogenic bacteria. These results indicate that Fla*_Las_* acts as a PAMP and may play an important role in triggering host plant resistance to the HLB bacteria.

## Introduction

Huanglongbing (HLB, also known as citrus greening) is a devastating disease of citrus. From the first documentation in early 20^th^ century China [1] to the recent findings in São Paulo, Brazil [2], and Florida, Texas and California in the USA [3], this newly emerging and century-old disease has been a major problem for citrus production, especially in major citrus-producing countries such as China, Brazil and the USA [4], [5]. The prevalent species of HLB bacteria, ‘*Candidatus* Liberibacter asiaticus’ (Las) is a Gram-negative bacterium with a significantly reduced genome (1.23 Mb) [6]. As an insect-transmitted and obligate plant pathogen, Las attacks all citrus species and citrus hybrids in the genus of *Citrus*, causing a systemic disease by residing in the phloem of the plant hosts [7], [8], [9]. Although there is no highly resistant cultivar, some resistance or field tolerance to HLB within citrus and citrus relatives has been described [7], [8], [9]. A wide range of responses were observed following graft-transmission of Las to 30 different genotypes of citrus trees [10]. However, different isolates of Las cause different levels of disease in citrus cultivars [11].

Due to Las infection, photoassimilate export from source leaves is delayed and symplastic transport into sieve tubes is impeded in the HLB-affected leaves [12]. Las bacterial populations are unevenly distributed within the sieve tubes, and multiple pockets of necrotic phloem are found throughout the vascular system with massive accumulations of starch and callose depositions [13]. In the early stages of HLB infection, degenerative changes include the swelling of the middle lamella between cell walls surrounding sieve elements and the deposition of amorphous callose in the sieve elements [7]. The impairment of photoassimilate export leads to diverse symptoms, including blotchy-mottle on leaves, yellow shoots and tree decline [13].

In response to a pathogen attack, multiple defense mechanisms are triggered in the host plants, including basal defense and gene-for-gene resistance. The plants basal defense is best exemplified by the recognition of pathogen-associated molecular patterns (PAMPs) or microbe-associated molecular patterns (MAMPs) by pathogen recognition receptors (PRRs) [14], [15]. The highly conserved N-terminal domain of flagellin, flg22, is characterized as a plant bacterial PAMP. The leucine-rich repeat flagellin-sensitive-2 (FLS2) is responsible for the perception of the bacterial flg22 in Arabidopsis. FLS2 orthologs have also been identified in *Nicotiana benthamiana* [16] tomato [17] and rice plants [18], based on the requirement of flg22 perception. The perception requires the interaction of FLS2 with a receptor kinase like BAK1 to form a functional FLS2/BAK1 complex. This event is followed by reactive oxygen species production, callose deposition, activation of a downstream mitogen-activated protein kinase pathway, and upregulation of a class of WRKY transcription factors [14], [15].

The PAMP activity of flg22 has been elucidated in a number of plant bacteria, including *Pseudomonas* and *Xanthomonas* [19], [20]. Although Las has a significantly reduced genome, it retains the entire flagellum-encoding gene cluster resembling those found in other closely-related members of the *Rhizobiaceae* [6]. Since Las encodes a flagellin and hook-associated protein (Fla) that contains a conserved flg22-like domain, the objectives of this study are to examine the PAMP activity and intrinsic filament production of the Las flagellin in alternate hosts and to explore the potential role of basal defense in the host plants’ resistance to the HLB bacteria.

## Results

### Sequence Analysis of the Putative Las Flagellin Gene

**Figure 1:**
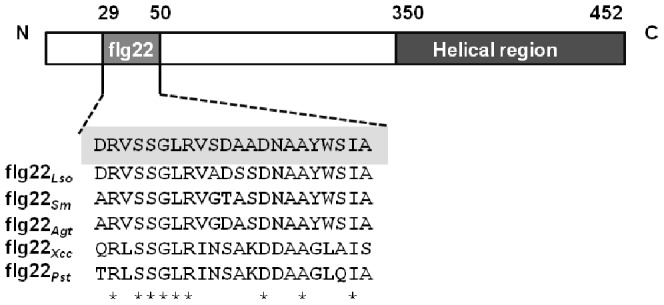
Schematic structure of the ‘*Candidatus* Liberibacter asiaticus’ flagellin domain sharing the conserved flg22 amino acid sequence. The localization of flg22 from position 29 to 50 at the N-terminus, and the helical region from position 350 to 452 at the C-terminus of the flagellin protein are shadowed. *Lso*, ‘*Ca.* Liberibacter solanacearum’; *Sm*, *Sinorhizobium meliloti* Rm1021; *Agt*, *Agrobacterium tumefaciens*; *Xcc, Xanthomonas citri* subsp. *citri*; *Pst, Pseudomonas syringae* pv. *tabaci.* * indicates a conserved amino acid.

In the genome of Las psy62, a complete set of flagellar biosynthesis genes were revealed by sequence comparison with other closely related bacterial genomes [6]. The *fli*, *flg*, *flh* and *mot* genes are considered to be sufficient for flagellar production and these genes (a total of 30 genes) are mainly located discontinuously at three genetic loci, 271164–277204, 572909–584140 and 749696–766368 (Fig. S1). The first gene in the third genetic locus (Accession number CP001677.5, GI: 25404028) encodes a flagellin domain-containing protein (Fla). This protein has 452 amino acids, with a 22 amino acids conserved flagellin domain from positions 29 to 50 at the N terminus. A helical region is also found at the C terminus (Fig. 1). By comparison with the GenBank database, the Fla*_Las_* shares 43–44% identity with the FlaA, FlaB, FlaC and FlaD of *S. meliloti* 1021, while the conserved domain, designated as flg22*_Las_*, shares 86% identity with that of *A. tumefaciens* str. C58 and ‘*Ca.* L. solanacearum’ CLso-ZC1, and 82% identity with that of *S. meliloti* 1021 (Fig. 1).

### Las Flagellin Functions in Bacterial Flagellum Biosynthesis

**Figure 2:**
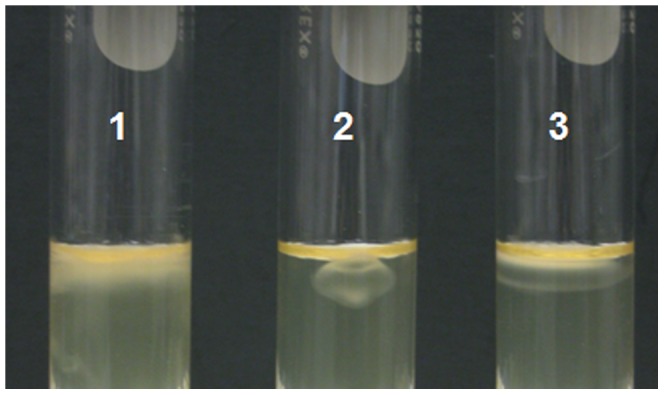
Complementation of *Sinorhizobium meliloti* flagellin (*fla*) mutant with ‘*Ca.* Liberibacter asiaticus' (Las) flagellin. The *S.* *meliloti* RU11/011 *fla* mutant strain has null mutant defects in all four *fla* genes and produces no flagellum. The Las flagellin gene was cloned into pBBR1MCS-5 to generate the constitutive expression construct pBB*fla* which was introduced into mutant RU11/011. 1, wild type RU11/001; 2, *fla* mutant RU11/011; 3, RU/pBB*fla*. The bacterial strains were cultured in LB liquid media overnight, and cell cultures were adjusted to OD_600_ = 0.5. An aliquot of 20 µL of cell suspensions were dropped onto the top surface in the test tubes. All the samples were placed in a 28°C incubator for 2 days. The results shown are representative of data from three independent replicates.

In order to understand the function of the Las flagellin in filament production, a *fla* mutant RU11/011 derived from *S. meliloti* was used since the Las bacterium has not been cultured *in vitro*. The RU11/011 mutant strain has null mutant defects in all four *fla* genes and produces no flagellum [21]. The Las flagellin gene was cloned into pBBR1MCS-5 to generate the constitutive expression construct pBB*fla*. When pBB*fla* was introduced into mutant RU11/011, the bacteria’s motility was partially restored. In test tubes, *fla* mutant RU11/011 cells grew in an aggregated shape, showing no motility (Fig. 2). The wild type cells were diffused into the soft media because of their motility, which made the media look turbid (Fig. 2). The pBB*fla* partially restored the bacteria’s motility; therefore, the bacterial cells diffused into the soft media to some extent (Fig. 2). These results indicate that Las encodes functional flagellin genes that may be involve in filament production, even though the flagellum has not yet been observed in Las.

### Las Flagellin Induced Cell Death in Tobacco Plant

**Figure 3:**
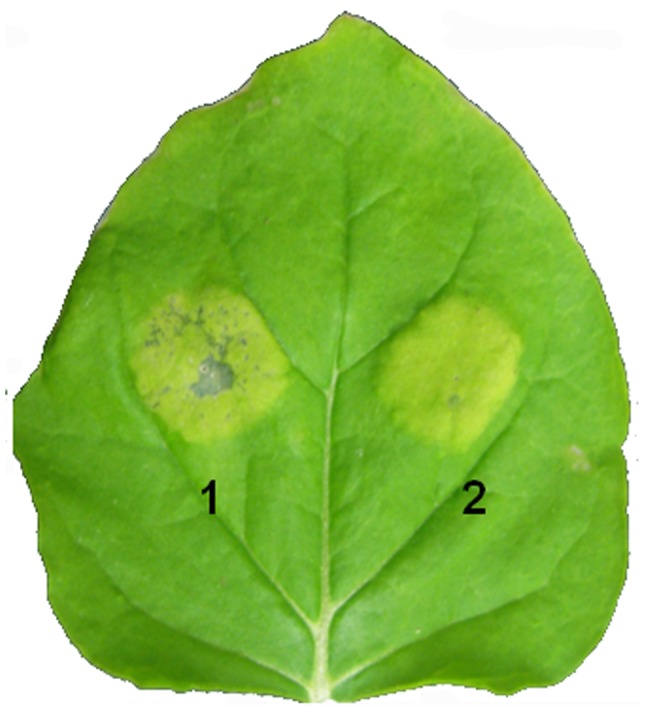
Cell death-like phenotypes on *Nicotiana benthamiana* leaves induced by *Ca.* Liberibacter asiaticus (Las) flagellin (*fla*). *Agrobacterium* (GV3101) mediated transient expression was carried out using pBINplus/ARS-2×35S vector. The full length of the Las *fla* gene was cloned into the binary vector pBINplus/ARS-2×35S in which the gene is under the control of the double 35S promoter. 1, pB*fla*; 2, BINplus/ARS-2×35S empty vector. Phenotypes were photographed at two weeks after infiltration inoculation.

For *in planta* expression of Las flagellin, the sequence covering the full length of CLIBASIA_02090 was PCR-amplified from the Las psy62 genome. The DNA fragment was inserted into the PVX vector pgR107 and transiently expressed in *N. benthamiana* by *Agrobacterium*-mediated transformation. Two weeks after infiltration, the inoculation zone became chlorotic, indicating weak cell necrosis (data not show). To confirm the induction of cell death by Las, CLIBASIA_02090 was additionally cloned into a binary vector, pBINPLUS/ARS-2×35S to generate pB*fla*, in which the gene was under the control of double 35S promoter. In this case, cell necrosis was clearly observed in the inoculation area infiltrated with the *Agrobacterium* carrying pB*fla* (Fig. 3), and it was evaluated by histochemical analysis using trypan blue staining (Fig. S2).

### Las Flagellin Functions as a PAMP

**Figure 4:**
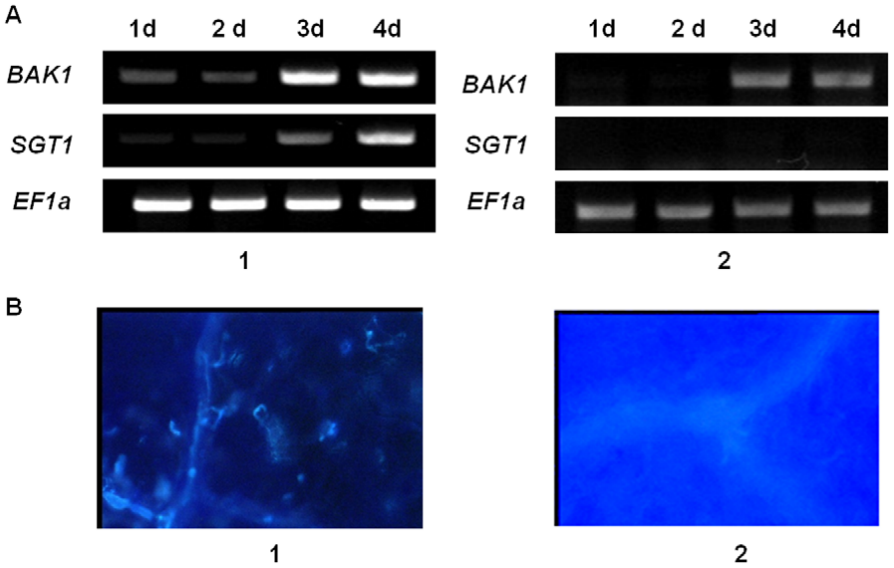
Pathogen-associated molecular pattern (PAMP) activity of ‘*Ca.* Liberibacter asiaticus’ (Las) flagellin (*fla*) in *Nicotiana benthamiana* leaves. BAK1 and SGT1 are co-receptors that interact with Fla through the leucine-rich repeat kinase-like receptor FLS2 to form a recognition complex. pV*fla* contains the full length of the Las *fla* gene inserted into the potato virus X (PVX) vector pgR107. **A,** RT-PCR detection of *BAK1* and *SGT1* expression. **B,** Callose deposition. 1, pV*fla*; and 2, PVX vector as control. EF1, *N. benthamiana* elongation factor 1 alpha, an internal quality control. The leaf samples were collected daily for four days post infiltration to detect *BAK1* and *SGT1* expression. For callose deposition, leaves were collected at 12 hours post infiltration. After staining in aniline blue solution, callose was visualized under UV epifluorescence.

Bacterial flagellin is perceived by a leucine-rich repeat kinase-like receptor FLS2 that interacts with BAK1 and SGT1 to form a recognition complex. To understand molecular events taking place in Las flagellin perception, RT-PCR was carried out to detect the expression of co-receptors BAK1 and SGT1 in tobacco plants. After tobacco was inoculated with GV3101/V*fla*, the expression levels were assessed daily for four days after infiltration (DAI). Low levels of *BAK1* and *SGT1* expression were observed at one and two DAI, but they were at increasingly higher levels at three and four DAI (Fig. 4 A1). In contrast, in the tobacco plant treated with the empty vector pgR107, no *SGT1* transcript was detected. The *BAK1* transcript was not detected at the first two DAI and only a low level of expression was detected at three and four DAI (Fig. 4 A2). This indicates that *Agrobacterium* GV3101 carrying the PVX empty vector induced a weak defense reaction in tobacco plants. Based on the gene expression pattern, callose deposition was examined at 12 hours after infiltration (HAI). The results indicated that Las flagellin induced callose deposition within 12 HAI in *N. benthamiana* (Fig. 4B).

### The S38 and D39 Amino Acids are Essential for PAMP Activity

**Figure 5:**
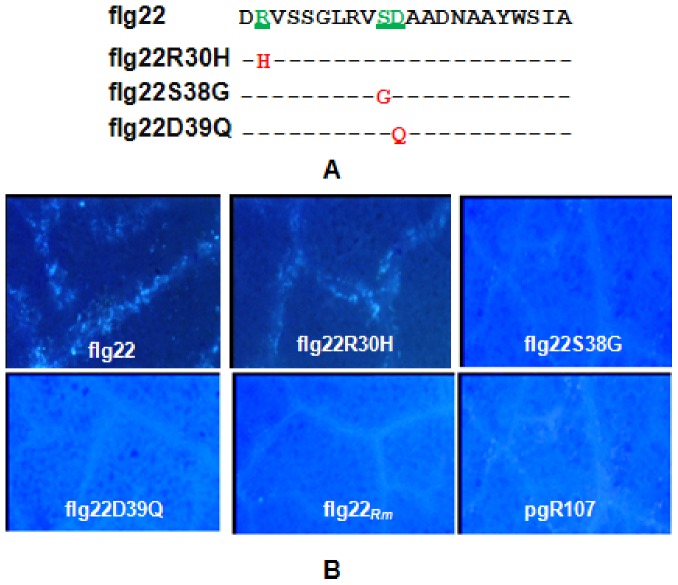
Impact of amino acid changes at positions R30, S38 and D39 in *Ca.* Liberibacter asiaticus (Las) flagellin’s conserved domain, flg22*_Las_*, on callose deposition. A, Amino acid changes made in flg22*_Las_*. The 30^th^, 38^th^ and 39^th^ amino acid residues were changed to histidine, glycine and glutamine by a primer extension strategy. **B,** Callose deposition. PCR products were inserted into the potato virus X vector pgR107 for Agro-infiltration. Callose deposition was visualized under UV epifluorescence at 12 hours after infiltration. The results shown are representative of data from four independent replicates.

The Las flg22 peptide shared 90% and 82% identity with the flagellin peptides from *A. tumefaciens* and *S. meliloti*, respectively. However, the flg22 peptides from *A. tumefaciens* and *S. meliloti* were reported to show no PAMP activity [22]. In comparison with the flg22 from *S. meliloti,* there are four amino acid differences found in flg22*_Las_*. The first two amino acid changes are aspartate (D29) and arginine (R30), at position 29 and 30, which are also the first two amino acids in flg22*_Las_*. The other two differences, serine (S38) and aspartate (D39), are in the middle of the flg22*_Las_* peptide (Fig. 5A). To investigate the importance of the divergent amino acids in flg22*_Las_*, site-directed mutagenesis by primer extension was employed to create site substitution mutants at R30, S38 and D39 in the flg22 region. The nucleotides of the desired mutants R30H, S38G and D39Q were individually inserted into the pgR107 vector for tobacco inoculation through infiltration. The flg22 of the *S. meliloti fla*B gene was inserted into pgR107 as a parallel control. At 12 HAI, the R30H mutant induced callose deposition in the infiltration area, while substitution mutants S38G and D39Q impaired the ability to induce callose deposition in tobacco leaves (Fig. 5B). This indicates that the residues at positions 38 and 39 in flg22 *_Las_* are essential for callose induction.

### Flg22*_Las_* Induces Callose Deposition without Cell Death *in planta*

**Figure 6:**
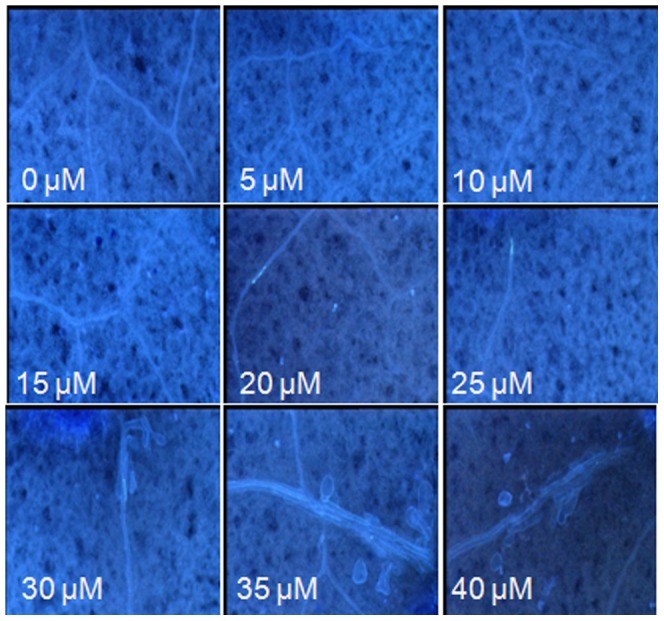
Minimum concentration of flg22 *_Las_* need for callose deposition. Synthetic peptide was diluted with double distilled water to a final concentrations of 5, 10, 15, 20, 25, 30 35 and 40 µM and infiltrated into tobacco leaves. Callose deposition was examined at 10 days later. The experiments were performed in three independent replicates.

To verify the minimum concentration of flg22*_Las_* necessary for callose induction, a synthetic flg22*_Las_* peptide was dissolved in double distilled water to final concentrations of 5, 10, 15, 20, 25, 30, 35 and 40 µM. The solutions were filtrated into tobacco leaves with 1 ml needleless syringes. Callose deposition was assessed in the infiltration area of tobacco leaves at 10 days after infiltration. At the lower concentrations of 5, 10 and 15 µM flg22*_Las_* could not induce callose deposition, while at a concentration of 20 µM flg22*_Las_* showed a weak ability to induce callose production. As the concentration was increased from 25 µM to 40 µM more callose deposition was observed (Fig. 6). This suggests that the minimal concentration of flg22*_Las_* for callose induction is 20µM. In contrast to the full length protein of the Las flagellin, the synthetic flg22*_Las_* peptide induced callose deposition without cell death even at two weeks after infiltration (data not shown).

## Discussion

Due to the fastidious nature of the HLB bacteria, there is little information about the molecular host-pathogen interactions in the HLB complex. In this study, we demonstrated that Las encodes a functional flagellin that acts as a PAMP, by expressing the Las *fla* gene in *S. meliloti* and through *Agrobacterium*-mediated transient expression. In addition we assessed the flg22*_Las_* peptide in its native and mutated forms in *N. benthamiana* to verify amino acids critical for host recognition.

The structure of the bacterial flagellum consists of three distinct parts: a basal body serving as a rotary motor, a rigid filament, and a flexible hook that couples the motor to the filament [23]. The filament is assembled from many flagellin subunits at the distal end of the flagellum. Bacteria with defective flagellin produce truncated flagella with reduced motility, or sometimes the bacteria are completely devoid of flagella. In *S. meliloti*, there are four flagellin-encoding genes. The critical *flaA* gene product combines with at least one of the other flagellin proteins to assemble a functional and/or truncated flagellar filament. Null mutations of all the four flagellin genes eliminate its flagellum formation [21]. In this study, Las flagellin was used to complement a *S. meliloti fla* mutant, and the motility was partially restored. This indicates that Las flagellin is functional and may be involved in filament production within Las. Although a Las flagellum has not yet been observed, our results imply that the Las bacterium may produce a functional flagellum in certain stages of its life as do other member of the order Rhizobiales, such as *Brucella meltinsis* that produces a polar flagellum transiently at the end of the exponential growth phase [24]. As with most other members of the Rhizobiacea, Las retains a complete set of flagellar biosynthesis genes despite having a significantly reduced genome (Fig. S1). The formation of the Las flagellum and its regulation may be critical for understanding the host-pathogen interactions in the HLB complex. Bacterial flagellins are frequently perceived by host recognition receptors to trigger defense responses. Flagellins from various bacteria have well conserved N- and C- termini and variable central portions. The conserved 22 amino acid peptide flg22 is near the N-terminus, and most flg22 peptides induce cell death. The flagellins from *P. syringae* pv. *tomato* and *glycinea* induce hypersensitive reactions in incompatible tobacco plants [19]. In *P. avenae,* the complete bacterial flagellin induced cell death in suspension-cultured rice cells, but flg22 alone did not induce cell death [14], [25]. In this study, transiently expressed Las flagellin induced cell death in tobacco plants, but the induction was weaker than that caused by the flagellin of other bacteria. The flg22*_Las_* peptide alone did not induce cell death. In addition, the minimum effective concentration of synthetic flg22*_Las_* was 20 µM, 10 to 20 fold higher than flg22 from *Pseudomonas* and *X. citri* subp. *citri* [26], [27]. Thus, the PAMP activity of flg22*_Las_* is apparently weaker than that of flg22 from other well-studied plant pathogenic bacteria.

In spite of the highly conserved amino acid sequences of flg22 from various bacteria, substitutions at certain positions resulted in a great attenuation or loss of PAMP activity. Replacement of aspartate at position 43 with valine or alanine abolished the ability of *P. syringae* pv. *tabaci* 6605 flg22 peptide to induce cell death [28]. The flg22 peptides from *A. tumefaciens* and *S. meliloti* have already been reported to have no PAMP activity [21]. The conserved Las flagellin domain flg22 in the N-terminus showed PAMP activity when transiently expressed in tobacco. It induced callose deposition as well as the expression of the genes associated with PAMP perception. Using the genomic data it was possible to compare flg22*_Las_* with flg22*_Lso_* from the related species ‘*Ca.* Liberibacter solanacearum’ CLso-ZC1 [29]; there are three divergent amino acids, one from serine to alanine and two from alanine to serine (Fig. 1). In future work, it will be interesting to investigate if the change from serine to alanine in flg22*_Lso_* has any effect on PAMP activity.

The perception of bacterial flagellin requires the LRR receptor kinase FLS2 and a co-receptor BAK1 to form a FLS2/BAK1 complex, which activates MAPK3 and MAPK6 and the downstream WRKY transcription factors to regulate gene expression [15], [27]. Even though SGT1 and BIK1 were reported to work as co-receptors, only the direct interaction between FLS2 and bacterial flagellin was fully elucidated [27]. The extracellular leucine-rich repeat and the kinase activity domains are both required for flagellin binding and subsequent signaling [30]. Thus, the interaction or recognition between FLS2 and flagellin is a pivotal step that determines whether flagellin can induce a defense reaction in plants. We have demonstrated that Fla*_Las_* acts as a PAMP in tobacco plants. We also found the flg22*_Las_* triggered different degrees of PAMP activity in different citrus species/cultivar (data not shown). The interaction of flg22*_Las_* with the corresponding FLS2 receptor in citrus plants is under investigation.

To provide protection from a pathogen attack, host plants employ PAMP-triggered immunity (PTI) and effector-triggered immunity (ETI) to recognize the intruders and activate a battery of defenses. PTI is the first layer of plant immunity, which restricts pathogen colonization. ETI is the second layer mediated by the recognition between a bacterial Avr protein and plant resistance (R) protein, resulting in the hypersensitive response or another resistance interaction [15]. In citrus canker caused by *X. citri* subsp. c*itri*, both PTI and ETI are employed by hosts to suppress the causal agent [27], [31]. However, in Las psy62, there is neither an Avr protein nor the type III secretion system responsible for delivering Avr protein effectors, and ETI does not appear to exist in the Las-citrus interaction. It is possible that PTI is the sole protective layer used by citrus plants to interfere with Las colonization and infection. As an intracellular bacterial pathogen, Las evolution may have reduced its modes for triggering host defense responses, which may explain why all genotypes display some degree of susceptibility and no strong resistance occurs in any citrus tested.

## Materials and Methods

### Bacteria and Plant Materials

The bacterial strains and plasmids used in this study are listed in Table 1. The *Escherichia coli* strains were grown at 37°C in Luria-Bertani (LB) medium. The *Agrobacterium tumefaciens* and *Sinorhizobium meliloti* strains were grown at 28°C in TY and LB/MC media, respectively [33], [37]. When required, antibiotics were added to the medium at the final concentrations: kanamycin, 50 µg/mL; neomycin, 50 µg/mL; streptomycin, 200 µg/mL; and gentamicin, 50 µg/mL.

**Table 1:** The strains and plasmids used in this study.

Strain or plasmid	Property	Resource
**Strains**		
*Agrobacterium tumefaciens*		
GV3101	Rif^r^, with Ti plasmid pMP90	[32]
GV3101/V*fla*	Rif^r^Km^r^, GV3101 carrying pV*fla*	This study
GV3101/B*fla*	Rif^r^Km^r^, GV3101 carrying pB*fla*	This study
GV3101/PVX	Rif^r^Km^r^, GV3101 carrying empty PVX vector pgR107	This study
GV3101/PBIN	Rif^r^Km^r^, GV3101 carrying empty binary vector pBINplus/ARS-2×35S	This study
GV3101/VFLG	Rif^r^Km^r^, GV3101 carrying Pvflg	This study
GV3101/VR30H	Rif^r^Km^r^, GV3101 carrying pVR30H	This study
GV3101/VS38G	Rif^r^Km^r^, GV3101 carrying pVS38G	This study
GV3101/VD39Q	Rif^r^Km^r^, GV3101 carrying pVD39Q	This study
*Sinorhizobium meliloti*		
RU11/001	Sm^r^ spontaneous streptomycin-resistant wild-type strain	[32]
RU11/011	Sm^r^ Nm^r^, *fla* null mutant derived from RU11/001, Δ*flaA* Δ*flaB* Δ*flaC* Δ*flaD*	[32]
RU/PBB*fla*	Sm^r^Nm^r^Gm^r^, RU11/011 carrying pBB*fla*	This study
*Escherichia coli*		
DH5α	F^−^ ϕ80d*lacZ*ΔM15Δ(*lacZYA-argF*)U169 *endA1 deoR recA1 hsdR17*(r_K_^−^ m_K_^+^) *phoA supE44 λ^−^ thi-l gyrA96 relA1*	Clontech
**Plasmid**		
pgR107	Km^r^, PVX vector with *Cla*I-*Sma*I-*Sal*I sites,	[33]
pV*fla*	Km^r^, Las Flagellin gene in pgR107,	This study
pBINplus/ARS-2×35S	Km^r^, a pBINPLUS/ARS binary vector derivative with double 35S promoter	[34]
pB*fla*	Km^r^, Las Flagellin gene in pBINplus/ARS-2×35S	This study
pBBR1MCS-5	Gm^r^, 5.1-kb broad-host range plasmid, *lac*Z	[35]
pBB*fla*	Gm^r^, Las flagellin gene in pBBR1MCS-5	This study
pRK600	Cm^r^, pRK2013 Nm^r^::Tn9	[36]
pVFLG	Km^r^, 66-bp fragment coding wild type flg22*_Las_* in pgR107	This study
pVR30H	Km^r^, 66-bp fragment coding flg22*_Las_* with change at position 30 from arginine to histidine in pgR107	This study
pVS38G	Km^r^, 66-bp fragment coding flg22*_Las_* with change at position 38 from serine to glycine in pgR107	This study
pVD39Q	Km^r^, 66-bp fragment coding flg22*_Las_* with change at position 39 from aspartate to glutamine in pgR107	This study

*N. benthamiana* seeds were stored at 4°C for 2 days prior to germination. Subsequently the seeds were germinated in chambers programmed for cycles of 16 h light and 8 h dark at 26°C. The seedlings were then transferred into Fafard 4P mix soil in plastic containers and grown in the greenhouse under controlled conditions.

### DNA Manipulation

DNA extraction, restriction enzyme digestion and polymerase chain reaction (PCR) were performed according to standard procedures [38]. The PCR primers in this report are listed in Table 2.

**Table 2:** Primers used in this study.

Primers	Primer Sequence (5′→3′)	Description
FLA-F	CC*CCCGGG^#^*ACAAGTAGCAATGACTAGTAT (*Xma*I)	To amplify a 1409-bp DNA fragment encompassing the 1359-bp Las flagellin gene of CLIBASIA_02090
FLA-R	TCC*CCCGGG^#^*CAAAAAACGGTTCTTCAAGCACAT (*Xma*I)	
P107-F	AATCACAGTGTTGGCTTGC	130-bp upstream of *Xma*I site in pgR107 vector for clone screening
N-F	TTA*CCCGGG^#^*ATGGATCGCGTTTCTTCAGGA	To amplify the Las wild type flg22 encoding sequence when combined with primer NS-R
NS2-F	TTA*CCCGGG^#^*ATGGAT**CAT**GTTTCTTCAGGATTACGCGTTTC	To produce a coding sequence with an amino acid change at position 30 from arginine to histidine when combined with NS-R
NS3-F	TTA*CCCGGG^#^*ATGGATCGCGTTTCTTCAGGATTACGCGTT**GGT** *GATGCTGCTCATAATGCA	To produce a coding sequence with an amino acid change at position 38 from serine to glycine when combined with primer NS-R
NS4-F	TTA*CCCGGG*ATGGATCGCGTTTCTTCAGGATTACGCGTTTCT**CAG** *GCTGATAATGCA	To produce a coding sequence with an amino acid change at position 39 from aspartate to glutamine when combined with primer NS-R
NS-R	TTT*CCCGGG^#^*CTATTTTGAAGATCCTAGGCCTATTGCA	The reverse primer used to produce transient expressing flg22 in the PVX vector
Smflg-F	TTT*CCCGGG^#^*ATGCAGGCGCATGTCTCCTCCG	To amplify a 66-bp DNA fragment encoding the flg22 in *flaB* gene in *S. meliloti*
Smflg-R	TTT*CCCGGG^#^*CTACATATTGTCGGAGCGCATGGTGGTC	
BAK1-F	TGAACGGTTGCTTGTTTATCCATAT	To amplify a 560-bp fragment of the *N. benthamiana BAK1* gene for RT-PCR detection
BAK1-R	CAGAGAAGAGCTACCCGAATAAG	
SGT1-F	CAGAGGAGGTGGTGGTGACTATA	To amplify a 541-bp fragment of the *N. benthamiana SGT1* gene for RT-PCR detection
SGT1-R	GGAGGGCTTCCTTCGACCTTCTTTG	
EF1a-F	TTGCCTTGTGGAAGTTTGAGAC	To amplify a 574-bp fragment of the *N. benthamiana EF1á* gene
EF1a-R	CATACCAGGCTTGAGGACACCAGTT	

#Restriction enzyme *Xma*I sites are in italics and underlined.

* Point mutation sites are in bold and underlined in primer sequence NS2-F, NS3-F and NS4-F.

### Transient Expression of the Las *Fla* Gene in *N. benthamiana*

In the Las str. psy62 genome, CLIBASIA_02090 encodes a flagellin domain-containing protein [6]. Primers Fla-F and Fla-R were used to amplify the full length of this gene by using DNA extracted from Las-infected citrus as a template (Table 2). The PCR product was inserted into *Xma*I site of potato virus X (PVX) vector pgR107, resulting in pV*fla*. To isolate the clones with the appropriate insertion orientation, primer P107-F was designed according to the nucleotide sequence of the pgR107 vector (Table 2). This primer was 130 bp upstream of the *Xma*I site and was combined with the reverse primer Fla-R for PCR screening. The Las *Fla* gene was additionally cloned into binary vector pBINplus/ARS-2×35S, in which the gene was under the control of the double 35S promoter [34]. In this case, the *Fla* gene was cut from pV*fla* and ligated into the *Xma*I site of pBINplus/ARS-2×35S. The recombinants were digested with *Hind*III to confirm the appropriate orientation insertion, which produced a 1.9 kb DNA fragment. This construct was designated as pB*fla*. If the foreign fragment was inserted in a reverse orientation, the recombinant would produce a 1.1 kb DNA fragment after *Hind*III digestion. After confirmation by sequencing, the recombinant constructs pV*fla* and pB*fla* with appropriate insertion orientations were transformed into *A. tumefaciens* GV3101 by a freeze-thaw method [39].

GV3101/V*fla* and GV3101/B*fla* were cultured in 2 mL of LB medium until they reached stationary phase. Fifty microliters of cell cultures were inoculated into 50 mL of fresh LB liquid medium, and grown until the OD_600_ value reached about 0.6. The cultures were centrifuged for 10 min at 2250 g, and re-suspended in 5 mL of Agromix (10 mM MgCl_2_, 10 mM MES, and 100 µM acetosyringone). After the suspension stood at room temperature for 1 to 2 h, the OD_600_ value was adjusted to the optical density of 2.0 with Agromix. The final cell suspension was infiltrated into 4 week-old *N. benthamiana* leaves with a 1 mL needleless syringe [33].

### Complementation of *S. meliloti fla* Mutant with the Las *Fla*

The opening reading frame of Las *Fla* was excised from pV*fla* by *Xma*I and ligated into cosmid pBBR1MCS-5 [34]. The appropriately oriented recombinant was confirmed by digestion with *Hind*III, and designated as pBB*fla*. Plasmid pBB*fla* was introduced into *S. meliloti fla* mutant RU11/011 by conjugation employing pRK600 as the mobilization vector [37]. Motility assays were conducted by dropping three microliter cells of each strain onto 0.3% agar LB/MC tubes. Tubes were photographed after 48 h incubation at room temperature [40]. This experiment was carried out with three to five replicates and repeated at least three times.

### Substitution Constructs

To obtain an expression construct of wild type flg22 *_Las_* in PVX, the 66-bp encoding sequence for the 22 conserved amino acids, flg22*_Las_*, was PCR-amplified by using primers NS-F and NS-R. The start codon ATG and stop codon TAG were introduced into the 5′ terminus of forward and reverse primers, respectively. Three other oligonucleotides NS2-F, NS3-F and NS4-F were combined with primer NS-R to produce the desired point mutations in flg22 *_Las_* (Table 2). Primer NS2-F was used to obtain the expression construct with an amino acid change at position 30 from arginine (R) to histidine (H). Primer NS3-F and NS4-F were used to make changes at position 38 from serine (S) to glycine (G) and at position 39 from aspartate (D) to glutamine (Q), respectively. The nucleotide sequence encoding flg22 of FlaB from *S. meliloti* was also PCR-amplified as a parallel control (Table 2). PCR products were ligated into the *Xma*I site of the pgR107 vector for transient expression in tobacco as described above. The experiments were repeated three times.

### Reverse Transcription PCR (RT-PCR)

RT-PCR was carried out to detect the transcription of *BAK1*and *SGT1*, which are involved in flagellin perception in tobacco. *N. benthamiana* elongation factor 1 alpha was used as an internal quality control. Primers used in the RT-PCR experiments are listed in Table 2. *N. benthamiana* leaves were harvested at 1, 2, 3 and 4 days after infiltration. After grinding to a powder in liquid nitrogen, RNA was extracted using 1 mL of Trizol reagent (Sigma-Aldrich, St. Louis, MO) per 100 mg of fresh tissue. The reverse transcriptase reactions were performed with 1 µg of total RNA using SuperScript® III Reverse Transcriptase (Invitrogen, Life Technologies, Carlsbad, CA) for 60 min at 50°C. Aliquots of the reverse transcription reactions (2.5% of total volume) were used as templates in 50 µL PCR reactions performed with 2.5 units of Taq polymerase (New England Biolab, Beverly, MA) per reaction. PCR was run for 33 cycles with an annealing temperature of 55°C. The PCR products were separated by electrophoresis in agarose gels stained with ethidium bromide. The experiments were repeated three times.

### Callose Deposition

To visualize callose deposition, leaf samples were treated and stained as described by Ausubel and Dewdney [41]. Briefly, the tissues were cleared and dehydrated with 100% ethanol. Cleared leaves were transferred sequentially to 50% ethanol and 67 mM K_2_HPO_4_ (pH 12), and then stained in 0.01% aniline blue in 67 mM K_2_HPO_4_ (pH 12) for 1 h at room temperature. Stained material was equilibrated in 50% glycerol and examined using ultraviolet epifluorescence (LEICA DMR microscope, Leica Wetzlar, Germany).

### Trypan Blue Staining

Cell death was evaluated by histochemical analysis using trypan blue [42]. Sample tissues were cleared and dehydrated with 100% ethanol in a boiling water bath for 2–3 min, covered with an alcoholic lactophenol trypan blue mixture (30 mL ethanol, 10 g phenol, 10 mL water, 10 mL glycerol, 10 mL lactic acid, and 20 mg trypan blue) at room temperature for 5 h and transferred into a chloral hydrate solution (2.5 g/mL). If necessary, chloral hydrate solution incubations were repeated several times to reduce the background. Samples were equilibrated in 50% glycerol and photographed with a digital camera.

### Peptide Synthesis and Infiltration

The peptide of flg22*_Las_* (DRVSSGLRVSDAADNAAYWSIA) was synthesized by LifeTein LLC (South Plainfield, NJ, USA) with a molecular weight of 2324.51. The synthetic peptide was diluted with double distilled water to final concentrations of 5, 10, 15, 20, 25, 30, 35 and 40 µM. At 10 days after infiltration into tobacco leaves, callose depositions were assessed following aniline staining.

## Supporting Information

Figure S1 **Localization and schematic features of three flagellar gene clusters in the** ***Candidatus*** **Liberibacter asiaticus Psy62 genome.** All the unlabelled genes are either hypothetical or non-flagellar genes. The flagellin domain-containing protein gene (*Fla*) is located at the beginning of the third gene cluster.
(TIF)

Figure S2 **Cell death in a tobacco leaf induced by** ***Agrobacterium*** **-mediated transient-expression of the** ***Candidatus*** **Liberibacter asiaticus (Las) flagellin (** ***fla*** **) illustrated by trypan blue staining.** 1, pBINplus/ARS-2×35S empty vector; 2, pB*fla*: pBINplus/ARS-2×35S containing the opening reading frame of Las *fla*. Leaf samples were observed 15 days after infiltration.
(TIF)
