# Supplementary figures and images for: The Destructive Citrus Pathogen, ‘Candidatus Liberibacter asiaticus’ Encodes a Functional Flagellin Characteristic of a Pathogen-Associated Molecular Pattern
Source: PLoS One. 2012 Sep 28;7(9):e46447. doi: 10.1371/journal.pone.0046447 (PMC3460909; doi:10.1371/journal.pone.0046447)

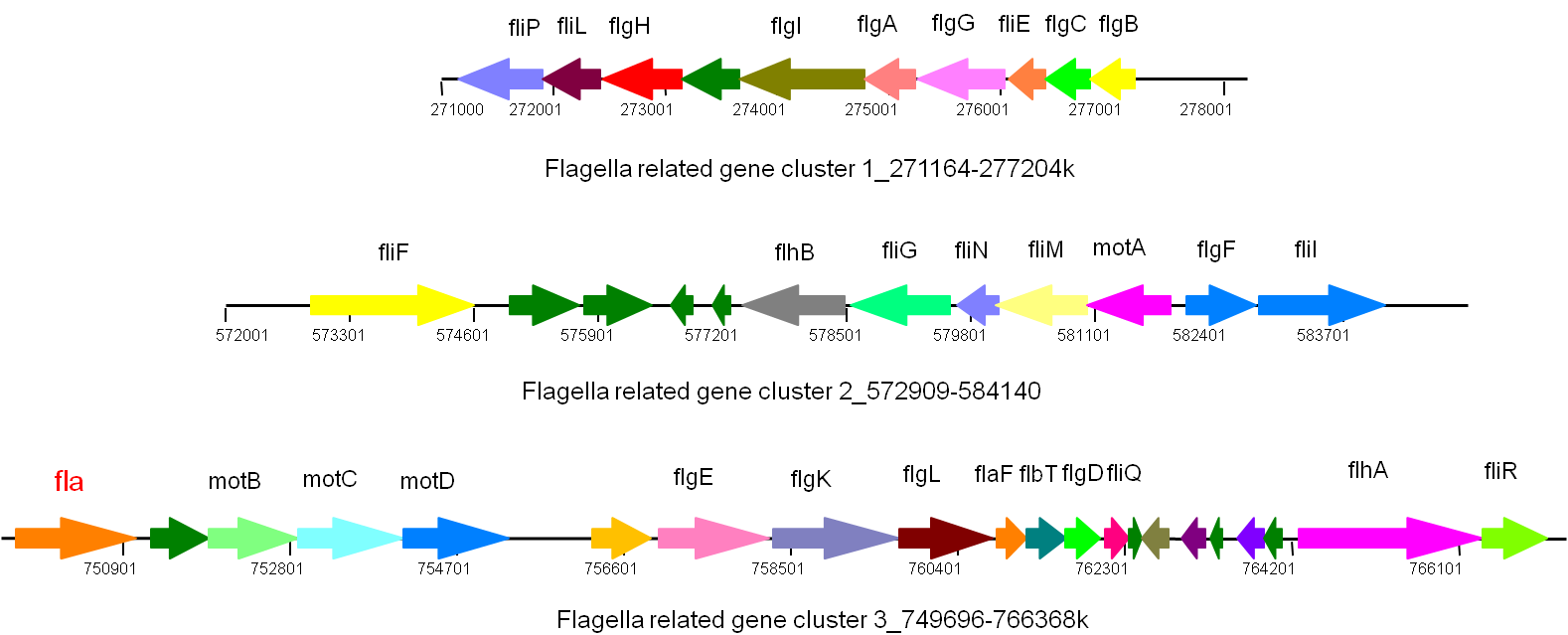

Supplement: Figure S1 — Localization and schematic features of three flagellar gene clusters in the Candidatus Liberibacter asiaticus Psy62 genome. All the unlabelled genes are either hypothetical or non-flagellar genes. The flagellin domain-containing protein gene (Fla) is located at the beginning of the third gene cluster. (TIF) [file pone.0046447.s001.tif]

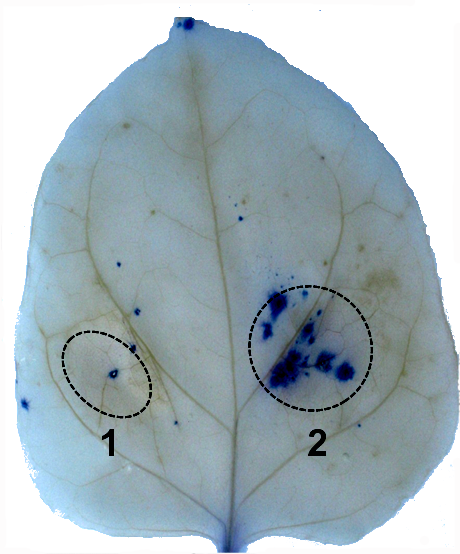

Supplement: Figure S2 — Cell death in a tobacco leaf induced by Agrobacterium -mediated transient-expression of the Candidatus Liberibacter asiaticus (Las) flagellin ( fla ) illustrated by trypan blue staining. 1, pBINplus/ARS-2×35S empty vector; 2, pBfla: pBINplus/ARS-2×35S containing the opening reading frame of Las fla. Leaf samples were observed 15 days after infiltration. (TIF) [file pone.0046447.s002.tif]
